# Solitary Celiac Lymph Node Metastasis Has a Better Long-Term Survival Compared With Solitary Mediastinal Lymph Node Metastasis in Esophagectomy of Esophageal Squamous Cell Cancer: A Propensity Score Matching Analysis

**DOI:** 10.3389/fonc.2022.834552

**Published:** 2022-03-11

**Authors:** Kun-Kun Li, Tao Bao, Ying-Jian Wang, Xiao-Long Zhao, Jiang Long, Xian-Feng Xie, Wei Guo

**Affiliations:** Department of Thoracic Surgery, Daping Hospital, Army Medical University, Chongqing, China

**Keywords:** esophageal squamous cell carcinoma, propensity score matching, minimally invasive esophagectomy, lymph node metastasis, long-term survival

## Abstract

**Background:**

The prognostic benefit of extensive lymphadenectomy remains controversial in esophageal squamous cell carcinoma (ESCC). The purpose of this retrospective study was to investigate the potential effect of solitary mediastinal (SM) lymph node metastasis and solitary celiac (SC) lymph node metastasis on the short- and long-term outcomes for patients who underwent minimally invasive McKeown esophagectomy.

**Methods:**

From September 2009 to December 2020, a total of 934 cases were diagnosed with ESCC and underwent minimally invasive McKeown esophagectomy in our department; 223 cases met the inclusion and exclusion criteria. Propensity score matching (PSM) was utilized to contrast the postoperative results and long-term survival of Group 1 (SM) and Group 2 (SC). Univariate and multivariate Cox proportional hazards regression analyses were used on possible predictors of survival.

**Results:**

One hundred forty-seven patients were available for outcome comparison after PSM. The postoperative results were not significantly different between the two groups. In terms of long-term survival, the 5-year disease-free survival (DFS) was 37.6% and 57.3% (*p* = 0.191) and 5-year disease-specific survival (DSS) was 39.7% and 68.4% (*p* = 0.028) for Group 1 (SM) and Group 2 (SC), respectively. Univariate and multivariate Cox proportional hazards regression analyses showed that body mass index (BMI), pathologic stage (pStage), and SC/SM grouping had significant hazard ratios (HRs), which suggested that SC is associated with better DSS.

**Conclusion:**

This cohort study showed that SC lymph node metastasis has a better long-term survival compared with SM lymph node metastasis in esophagectomy of ESCC. The results challenge the current understanding and need confirmation in further research.

## Introduction

Esophageal cancer (EC) is one of the most lethal and most aggressive cancers that ranks seventh in terms of incidence and sixth in mortality overall globally (1). Esophageal squamous cell carcinoma (ESCC) is the main pathological type of EC and carries an enormous burden in China, with a combined 5-year standardized relative survival of 20.9%–40.1% (2, 3). Advances in surgical techniques, perioperative care, and multidisciplinary synthetic therapy have increased the chance of curing localized disease, and the cumulative 5-year overall survival has reached 63% in resectable ESCC patients (4). Local recurrence and metastasis are the leading causes of death and can occur even in the early stages. The surgical techniques used for esophagectomy with lymphadenectomy are regarded as the cornerstone of treatment and are aimed toward achieving radical cure (5).

Since lymph node metastases of ESCC can primarily occur from the cervical to the abdominal field, a strategy for extended lymph node (LN) dissection has been established (6). The American Joint Committee on Cancer (AJCC) recommended the removal of 10 LNs for primary tumor stage (pT) tumors, 20 for pT2, and 30 for pT3–T4 ESCC (7, 8). LN stations based on the anatomical lymphatic spread is also useful for the assessment of prognosis (9). Thoracic surgeons believe that they can dissect the mediastinal LNs in a standard manner, but for celiac LNs (10), most of them are limited to dissect No. 16 and No. 17 stations; No. 18–20 stations were widely neglected (11) [the anatomical definitions of LN stations were based on AJCC, 8th edition (8)]. Here, we aimed to test whether solitary mediastinal (SM) lymph node metastasis and solitary celiac (SC) LN metastasis have a different effect on the short- and long-term outcomes for patients who underwent thoracoscopic–laparoscopic McKeown esophagectomy. We used a retrospective and comprehensive clinical data collection from a high-volume surgery center in China with one surgical team specialized in the mediastinal and celiac LN dissection of esophagectomy in ESCC.

## Patients and Methods

### Patients

From September 2009 to December 2020, a total of 934 cases were diagnosed with ESCC and accepted minimally invasive McKeown esophagectomy in our department (Department of Thoracic Surgery, Daping Hospital, Army Military Medical University Chongqing, China) by one surgical team (Pro. Guo). Patient data were obtained *via* retrospective chart assessment. The postoperative TNM stage was classified following the 8th edition of the AJCC staging protocol (8). Evaluation of the perioperative variables and assessment of the pertinent features of the patients were conducted. Postoperative death was considered as death within 30 days following surgery. All of the patients included in this evaluation provided written consent for surgery. The evaluation was approved by the Ethics Committee of Daping Hospital, Army Medical University.

The inclusion criteria were as follows: 1) histologically confirmed thoracic ESCC, 2) those who underwent minimally invasive McKeown esophagectomy and had complete clinicopathological data, 3) R0 resection of tumor and LNs, 3) positive LNs in solitary celiac stations or solitary mediastinal stations.

The exclusion criteria were as follows: 1) non-curative (R1 or R2) resection (tumor-free margin <1 mm), 2) loss to follow-up, 3) death due to causes not related to ESCC, 4) the number of LNs dissected <15, 5) grouping of LNs was not detailed.

**Figure 1:**
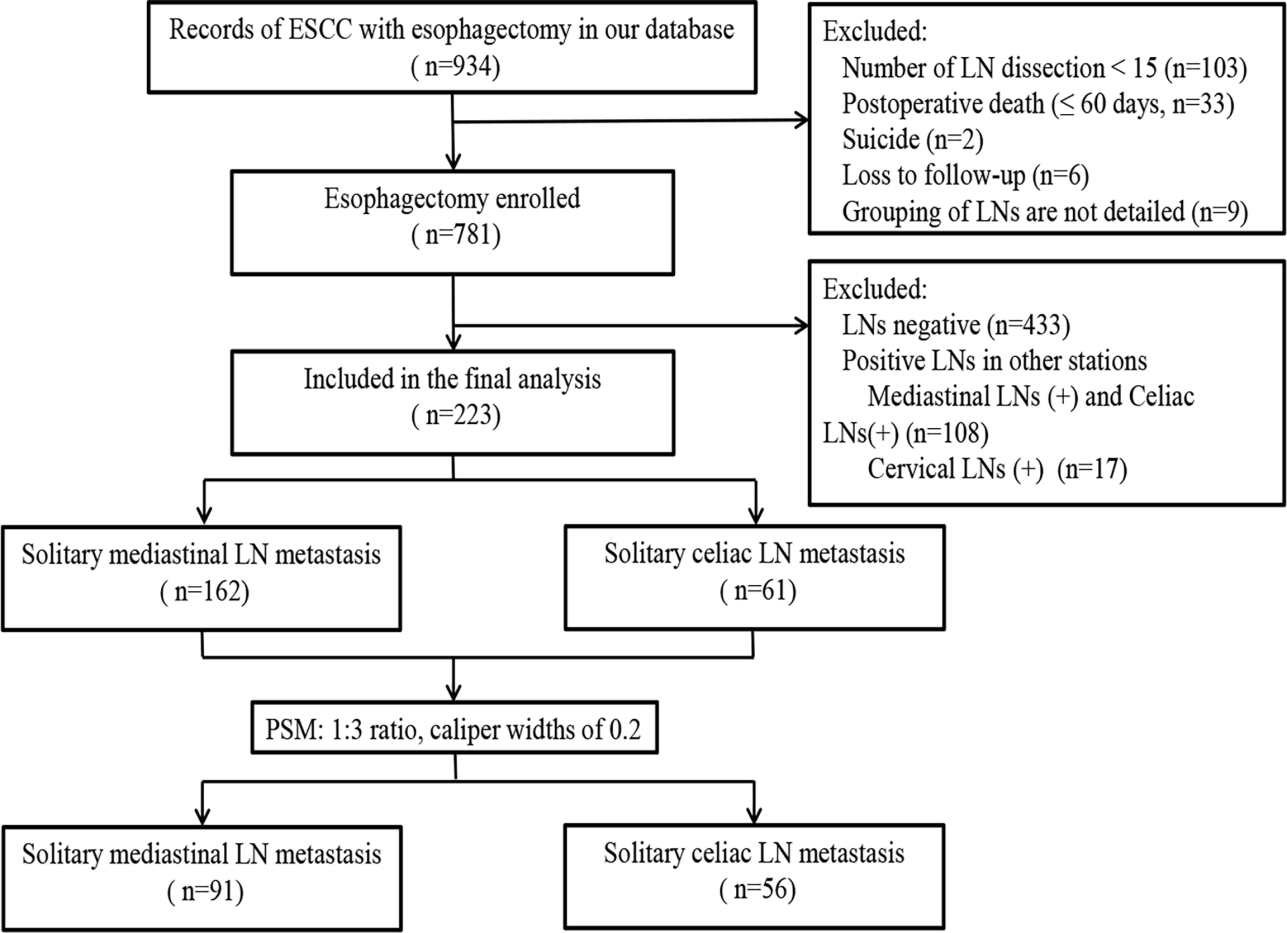
Flowchart of patient inclusion, allocation, and analysis. In this study, 223 patients were enrolled. After PSM, 147 patients were available for outcome comparison.

In this study, 223 cases were enrolled, and the flowchart of patient selection is shown in **Figure 1**. After propensity score matching (PSM), 147 patients were available for outcome comparison who were divided into Group 1 (SM) and Group 2 (SC).

### Surgical Procedures

The main surgical approach was thoracoscopic McKeown esophagectomy as previously described (12, 13). Briefly, the thoracic esophagus was shifted from the hilum and pericardium to the cervicothoracic area, mediastinal lymphadenectomy included but was not limited to stations of No. 2L, 2R, 7, 8U/M/L, 15. For the next stage, laparotomy or laparoscopy was performed with the patient in a supine position, the celiac lymph node dissection included at least No. 16, 17, 18, 19, 20. The preferred esophageal substitute was the gastric conduit, and a cervical esophagogastric anastomosis was created.

### Follow-Up

All of the patients were followed up in the outpatient clinic or *via* telephone contact after surgery, and follow-up information was obtained from all subjects included in the study until July 2021 or death. The follow-up duration ranged between 2.7 and 124.3 months (mean: 34.4 ± 28.3 months). Particular evaluations, including spiral computed tomography, barium swallow, and endoscopy, were conducted during the follow-up. The diagnosis of recurrence was established based on the pathologic or radiologic findings. Recurrences were classified as locoregional recurrences, distant metastasis, or death from ESCC. Disease-free survival (DFS) was defined as survival from surgery to the date of recurrence. Disease-specific survival (DSS) was defined as survival from surgery to the date of death due to ESCC.

### Statistical Analysis

Numerical data are presented as the mean ± standard deviation, and continuous and categorical variables were compared with the t-test and Fisher’s exact test or χ^2^ test, respectively. Univariate and multivariate Cox proportional hazards regression analyses were performed to identify potential prognostic factors. Survival curves were constructed using the Kaplan–Meier method, and the log-rank test was used to compare survival differences between the groups for each variable. Statistical analyses were performed by using SPSS statistical software, version 22.0 (IBM SPSS, Chicago, IL, USA). To minimize confounding effects caused by nonrandomized assignment, the postoperative outcomes of SM and SC groups were compared using PSM. PSM accounted for pT stage (tumor depth) and tumor location. Matching was performed in a blinded manner (1:3 ratio, caliper distance = 0.02) without replacement using the nearest method in the MATCHIT package (version 3.0.2) for R (version 4.1.1). A *p*-value <0.05 was considered statistically significant.

## Results

### Relevant Patient Characteristics Before and After Propensity Score Matching

The 223 patients included in this study consisted of 188 men and 35 women with an average age of 63.08 ± 8.26 years and a mean body mass index (BMI) of 22.09 ± 3.06. Fifty-six patients have no smoking history, and 30 patients received neoadjuvant chemotherapy or chemoradiotherapy. The baseline characteristics of the patients are shown in **Table 1**. Overall, there were no differences between the two groups except for pT stage and tumor location. There were more T3/T4 stage tumors (118/162, 72.8% vs. 32/61, 52.5%; *p* = 0.04) and in the upper thoracic esophagus (51/162, 31.5% vs. 1/61, 1.6%; *p* = 0.00) in Group 1 than in Group 2. After PSM (91 matched patients in Group 1 and 56 matched patients in Group 2), these findings were no longer statistically significant.

**Table 1:** Clinical and other relevant patient characteristics before and after propensity score matching.

Characteristics	Unmatched patients No. (%)		*p* value	Matched patients No. (%)		*p* value
	Group 1 (SM) (*n* = 162)	Group 2 (SC) (*n* = 61)		Group 1 (SM) (*n* = 91)	Group 2 (SC) (*n* = 56)	
Gender			0.56			0.15
Men	138 (85.2)	50 (82.0)		81 (89.0)	45 (80.4)	
Women	24 (14.8)	11 (18.0)		10 (11.0)	11 (19.6)	
Age (years)	62.94 ± 8.51	63.46 ± 7.61	0.68	62.20 ± 8.44	63.79 ± 7.56	0.25
Smoking history			0.20			0.19
No	37 (22.8)	19 (31.1)		19 (20.9)	17 (30.4)	
Yes	125 (77.2)	42 (68.9)		72 (79.1)	39 (69.6)	
BMI (kg/m^2^)	22.09 ± 2.97	22.09 ± 3.33	0.99	21.76 ± 2.79	22.14 ± 3.39	0.46
Neoadjuvant therapy			0.93			0.80
No	140 (86.4)	53 (86.9)		80 (87.9)	50 (89.3)	
Yes	22 (13.6)	8 (13.1)		11 (12.1)	6 (10.7)	
pT			0.04^*^			0.36
T0	0 (0.0)	1 (1.6)		Null	Null	
T1	16 (9.9)	10 (16.4)		12 (13.2)	8 (14.3)	
T2	28 (17.3)	18 (29.5)		18 (19.8)	18 (32.1)	
T3	99 (61.1)	27 (44.3)		54 (59.3)	27 (48.2)	
T4	19 (11.7)	5 (8.2)		7 (7.7)	3 (5.4)	
pN			0.67			0.36
N1	125 (77.2)	47 (77.0)		74 (81.3)	42 (75.0)	
N2	35 (21.6)	14 (23.0)		17 (18.7)	14 (25.0)	
N3	2 (1.2)	0 (0.0)		Null	Null	
Distant metastasis (M)			>0.99			>0.99
M0	162 (100.0)	61 (100.0)		91 (100.0)	56 (100.0)	
M1	0 (0.0)	0 (0.0)		0 (0.0)	0 (0.0)	
Tumor location			0.00^*^			0.17
Upper thoracic	51 (31.5)	1 (1.6)		3 (3.3)	1 (1.8)	
Middle thoracic	87 (53.7)	32 (52.5)		64 (70.3)	32 (57.1)	
Lower thoracic	24 (14.8)	28 (45.9)		24 (26.4)	23 (41.1)	
Tumor differentiation			0.14			0.48
Gx	3 (1.9)	3 (4.9)		3 (3.3)	1 (1.8)	
G1	8 (4.9)	5 (8.2)		6 (6.6)	5 (8.9)	
G2	105 (64.8)	40 (65.6)		51 (56.0)	37 (66.1)	
G3	46 (28.4)	13 (21.3)		31 (34.1)	13 (23.2)	
pStage			0.22			0.50
II	13 (8.0)	9 (14.8)		8 (8.8)	8 (14.3)	
III	138 (85.2)	50 (82.0)		81 (89.0)	46 (82.1)	
IV	11 (6.8)	2 (3.2)		2 (2.2)	2 (3.6)	

BMI, body mass index; T, tumor stage (depth of invasion); N, lymphatic dissemination stage [based on the AJCC (7), 8th edition; N0, no positive lymph nodes; N1, 1~2 positive lymph nodes; N2, 3~6 positive lymph nodes; N3, >6 positive lymph nodes].

^*^Statistically significant at p < 0.05.

### Surgical Outcomes and Postoperative Complications After Propensity Score Matching

Overall, as shown in **Table 2**, the average duration of surgery, intraoperative blood loss, number of retrieved nodes, and postoperative hospital stay were 233.40 ± 60.80 min, 167.82 ± 169.97 ml, 30.07 ± 11.40, and 16.12 ± 10.51 days, respectively, and the two groups did not significantly differ.

**Table 2:** Intraoperative data and postoperative complications after PSM.

Characteristics	Total (n = 147)	Group 1 (SM) (n = 91)	Group 2 (SC) (*n* = 56)	*p* value
Operation duration (min)	233.40 ± 60.80	238.98 ± 62.53	224.34 ± 57.26	0.16
Intraoperative blood loss (ml)	167.82 ± 169.97	170.11 ± 148.30	164.11 ± 201.68	0.84
Number of retrieved nodes	30.07 ± 11.40	28.90 ± 10.02	31.98 ± 13.22	0.11
Postoperative hospital stay (days)	16.12 ± 10.51	16.56 ± 10.48	15.39 ± 10.62	0.52
Postoperative complications				
Anastomotic complications				
Anastomotic leakage	28 (19.05)	19 (20.88)	9 (16.07)	0.47
Respiratory complications				
Pulmonary infection	10 (6.80)	6 (6.59)	4 (7.14)	0.90
ARDS	5 (3.40)	1 (1.09)	4 (7.14)	0.05
Pulmonary atelectasis	1 (0.68)	1 (1.09)	0 (0.00)	0.43
Pleural complications				
Pneumothorax	12 (8.16)	10 (6.80)	2 (3.57)	0.11
Pleural effusion	9 (6.12)	4 (4.39)	5 (8.92)	0.27
Chylothorax	2 (1.36)	1 (1.09)	1 (1.79)	0.73
Hemothorax	1 (0.68)	1 (1.09)	0 (0.00)	0.43
Others				
Wound infection	3 (2.04)	1 (1.09)	2 (3.57)	0.30
Vocal cord palsy	5 (3.40)	3 (3.29)	2 (3.57)	0.93
Seroperitoneum	1 (0.68)	1 (1.09)	0 (0.00)	0.43
Incisional hernia	1 (0.68)	1 (1.09)	0 (0.00)	0.43
Phlebothrombosis	1 (0.68)	0 (0.00)	1 (1.78)	0.20

PSM, propensity score matching; ARDS, Acute Respiratory Distress Syndrome.

The incidence of postoperative complication was 38.1% (56/147) (summarized in **Table 2**). Of note, the incidence of the six most common postoperative complications, i.e., anastomotic leakage (19/91, 20.88% vs. 9/56, 16.07%; *p* = 0.47), pneumothorax (10/91, 6.80% vs. 2/56, 3.57%; *p* = 0.11), pulmonary infection (6/91, 6.59% vs. 4/56, 7.14%; *p* = 0.90), pleural effusion (4/91, 4.39% vs. 5/56, 8.92%, *p* = 0.27), Acute Respiratory Distress Syndrome (ARDS) (1/91, 1.09% vs. 4/56,7.14%; *p* = 0.05), and vocal cord palsy (3/91, 3.29% vs. 2/56, 3.57%; *p* = 0.93) did not significantly differ between the two study groups.

### Recurrence and Survival After Propensity Score Matching

**Figure 2:**
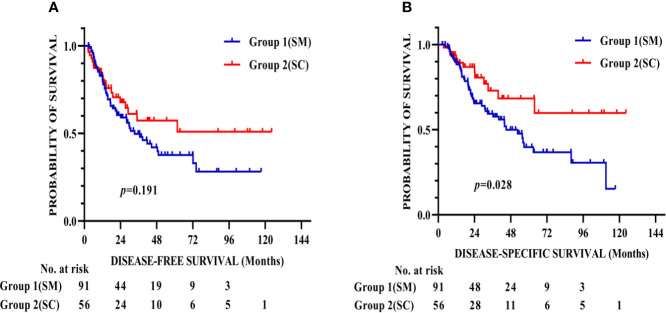
Kaplan–Meier survival curves for DFS **(A)** and DSS **(B)** of 147 patients after propensity score matching. The patients in Group 2 (SC) had better DSS than that in Group 1 (SM) (*p* = 0.028). DFS, Disease-free survival; DSS, Disease-specific survival.

**Figure 2** shows that the respective DFS rates of patients in Group 1 (SM) and Group 2 (SC) were 81.6% and 83% at 1 year, 49.8% and 57.3% at 3 years, and 37.6% and 57.3% at 5 years after surgery. The DSS rates were 89.6% and 91.8% at 1 year, 59.4% and 72.9% at 3 years, and 39.7% and 68.4% at 5 years after surgery in Group 1 (SM) and Group 2 (SC), respectively. The patients in Group 2 (SC) had better DSS than that in Group 1 (SM) (*p* = 0.028).

The prognostic factors for DSS are presented in **Table 3**. Univariate analysis showed that gender, age, smoking history, neoadjuvant therapy, pT stage, pathologic lymph node (pN) stage, tumor location, and tumor differentiation did not significantly influence DSS. However, BMI (*p* = 0.017), pStage (*p* = 0.006), and SC/SM grouping (*p* = 0.031) were associated with DSS. In addition, the findings were similar after multivariate Cox proportional hazards regression analysis. This analysis showed that BMI (*p* = 0.019), pStage (*p* = 0.013), and SC/SM grouping (*p* = 0.037) had significant HRs and were significantly correlated with DSS, which suggested that Group 1 (SC) is associated with better DSS.

**Table 3:** DSS differences after multivariate Cox proportional hazards regression analysis for the matched patients.

Variables	Univariate regression *p* value	Multivariate regression *p* value	Hazard ratio (95% CI for HR)
BMI (kg/m^2^)	0.017	0.019	0.888 (0.804–0.981)
pStage	0.006	0.013	3.637 (1.318–10.032)
SC and SM grouping	0.031	0.037	0.504 (0.265–0.961)
Gender	0.637		
Age (years)	0.260		
Smoking history	0.570		
Neoadjuvant therapy	0.780		
pT	0.065		
pN	0.582		
Tumor location	0.156		
Tumor differentiation	0.770		

DSS, disease-specific survival; SM, solitary mediastinal LN metastasis; SC, solitary celiac LN metastasis; LN, lymph node.

## Discussion

Over the past decade, the prognostic benefit of extensive lymphadenectomy was controversial. Some studies have found that a more extensive lymphadenectomy is associated with superior pathologic staging, and the overall survival continued to increase with increasing lymph node harvest (14–16). Others believe that extensive lymphadenectomy had the disadvantage of additional surgical trauma with greater surgical morbidity that may further worsen survival (17, 18). Although the current study did not provide any direct evidence supporting the extent of lymphadenectomy as a prognostic factor in ESCC, based on our experience, we found that 162 (162/781, 20.7%) patients have LN metastasis only in the mediastinum while the celiac LNs were negative, and 61 (61/781, 7.8%) patients have LN metastasis only in the celiac while the mediastinal LNs were negative. We naturally think about the long-term survival of these two groups.

The baseline characteristics of the patients showed that T stage and tumor location are the confounding factors in the evaluation of SC/SM grouping and long-term survival, and it is not feasible to randomize patients into numerous categories of lymphadenectomy; thus, the present study used PSM to minimize the effects caused by nonrandomized assignment. After PSM, T stage and tumor location did not significantly differ between the two groups, and the surgical outcomes and postoperative complications between the two groups were not significantly different either. In terms of long-term survival, the 5-year DFS was 37.6% and 57.3% (*p* = 0.191) and 5-year DSS was 39.7% and 68.4% (*p* = 0.028) for Group 1 (SM) and Group 2 (SC), respectively. From the Kaplan–Meier survival curve of DFS, although the log-rank test showed that there were no differences in these two groups, the tendency clearly presents that Group 2 (SC) has a better DFS compared with that of Group 1 (SM), and the small size of samples was considered to be the main reason. The DSS has a significant difference in these two groups and indicates that patients who have solitary celiac LN metastasis had significantly greater DSS than those who have solitary mediastinal LN metastasis. Univariate and multivariate Cox proportional hazards regression analysis determined that BMI, pStage, and SC/SM grouping are independent prognostic factors for long-term survival.

Our surgical team underwent standard thoracoscopic and laparoscopic training, hence minimizing the potential bias resulting from different surgical skills and experience. An additional strength of our study is that all enrolled patients underwent extensive lymphadenectomy, which provided the basis for accurate LN staging, researching of LNs and correlation with survival. The choice of cervical lymphadenectomy in esophagectomy is still controversial (19, 20). We prefer cervical ultrasound in upper-thoracic esophageal cancer; the cervical lymphadenectomy would be done if the ultrasound found enlargement of a cervical lymph node. The cases with positive cervical lymph nodes were excluded in this study, and the longitudinal and circumferential resection margins of the enrolled patients meet the clinical criteria as Migliore et al. (21) reported.

To the best of our knowledge, this is the first study to show the interesting finding that solitary celiac LN metastasis has a better long-term survival compared with solitary mediastinal lymph node metastasis post esophagectomy in ESCC. Furthermore, we could make some additional speculations. Firstly, the incidence of skip metastasis like SC is lower; the cancer cells are more likely to metastasize from mediastinal to cervical and then celiac in that order. Secondly, solitary celiac node metastasis is perhaps not the kind of distant metastasis as previously assumed, to have a worse prognosis, perhaps because there were more lymph node dissection in Group 2 (SC) than that in Group 1 (SM), and some studies found that the extent of lymph node involvement, including more positive or negative lymph nodes, is one of the most important prognostic factors to some extent (22–24). Thirdly, radical celiac lymph node dissection is beneficial for accurate LN staging and prognosis; hence, thoracic surgeons need to consider celiac LN dissection as important as mediastinal LN dissection.

Although PSM in this study was used to minimize the bias, the sample size that met the inclusion and exclusion criteria is small, and the results need to be further verified by large samples from homogeneous surgical procedures by multicenter. Additionally, most patients with solitary celiac positive LNs were lower-thoracic ESCC, and as reported by previous studies (25, 26), survival increased with a more distal location of cancer within the esophagus, which may be one of the potential biases of this study.

This cohort study showed that solitary celiac lymph node metastasis has a better long-term survival compared with solitary mediastinal lymph node metastasis in esophagectomy of ESCC. The results challenge the current understanding and need confirmation with further research.

## Data Availability Statement

The datasets generated and/or analyzed during the current study are not publicly available to protect patient confidentiality but are available from the corresponding author on reasonable request.

## Ethics Statement

The studies involving human participants were reviewed and approved by the Ethics Committee of Daping Hospital, Army Medical University. The patients/participants provided their written informed consent to participate in this study.

## Author Contributions

K-K L and TB contributed equally to this work and should be considered the first coauthors. K-K L, TB and WG conceived the study concept and participated in its design, data extraction, statistical analysis, and article drafting and editing. Y-J W and X-L Z recorded the follow-up data. JL and X-F X analyzed the data. All authors read and approved the final article.

## Funding

This work was supported by Chongqing medical scientific research project (the Joint project of Chongqing Health Commission and Science and Technology Bureau, 2022ZDXM017).

## Conflict of Interest

The authors declare that the research was conducted in the absence of any commercial or financial relationships that could be construed as a potential conflict of interest.

## Publisher’s Note

All claims expressed in this article are solely those of the authors and do not necessarily represent those of their affiliated organizations, or those of the publisher, the editors and the reviewers. Any product that may be evaluated in this article, or claim that may be made by its manufacturer, is not guaranteed or endorsed by the publisher.
